# Sex Differences in Oxycodone/Naloxone vs. Tapentadol in Chronic Non-Cancer Pain: An Observational Real-World Study

**DOI:** 10.3390/biomedicines10102468

**Published:** 2022-10-02

**Authors:** Jordi Barrachina, Cesar Margarit, Javier Muriel, Vicente López-Gil, Santiago López-Gil, Pura Ballester, Laura Mira-Lorente, Laura Agulló, Ana M. Peiró

**Affiliations:** 1 Neuropharmacology on Pain (NED), Alicante Institute for Health and Biomedical Research (ISABIAL-FISABIO Foundation), c/Pintor Baeza, 12, 03010 Alicante, Spain; 2 Pain Unit, Department of Health of Alicante—General Hospital, c/Pintor Baeza, 12, 03010 Alicante, Spain; 3 Occupational Observatory, Miguel Hernández University of Elche, Avda. de la Universidad s/n, 03202 Elche, Spain; 4 Department of Pharmacology, Paediatrics and Organic Chemistry, Miguel Hernández University of Elche, Crta. Nacional, N-332. s/n, 03505 Sant Joan, Spain; 5 Clinical Pharmacology Unit, Department of Health of Alicante—General Hospital, c/Pintor Baeza, 12, 03010 Alicante, Spain

**Keywords:** chronic pain, sex, tapentadol, oxycodone, bias

## Abstract

Despite the large body of research on sex differences in pain, there is a lack of translation to real-world pain management. Our aim was to analyse the sex differences in the analgesic response to oxycodone/naloxone (OXN) and tapentadol (TAP), in comparison with other opioids (OPO) commonly prescribed for chronic non-cancer pain (CNCP). An observational and cross-sectional study was conducted on ambulatory CNCP patients (*n* = 571). Sociodemographic, clinical (pain intensity, relief, and quality of life), safety (adverse events (AEs), adverse drug reactions), hospital frequentations and pharmacological (morphine equivalent daily dose (MEDD)) variables were collected. Multiple linear regressions were carried out to assess the association between sex and outcomes. Sex differences were observed, with lower female tolerability and higher hospital frequentation, especially in the OXN group (OR AEs report = 2.8 [1.8–4.4], *p* < 0.001). Here, females showed higher hospital use (23% hospital admission, 30% prescription change, *p* < 0.05), requiring a higher MEDD (127 ± 103 mg/day, *p* < 0.05), compared to OXN men. Regardless of the opioid group, CNCP women were significantly older than men (three years), with significantly higher benzodiazepine use (OR = 1.6 [1.1–2.3]), more constipation (OR = 1.34 [0.93–1.90]) and headache (OR = 1.45 [0.99–2.13]) AEs, than men who were more likely to refer sexual dysfunction (OR = 2.77 [1.53–5.01]), and loss of libido (OR = 1.93 [1.22–3.04]). Sex-differences were found related to poorer female drug tolerability and higher hospital resources, even worst in OXN female users. Other differences related to older female ages and benzodiazepine prescription, need to be further analysed from a gender perspective.

## 1. Introduction

There is increasing evidence to suggest that men and women differ in their analgesic responses [1] which could impact long-term quality of life differently [2,3]. Some biological mechanisms have been posited to explain these sex-related differences [4], due to a hormonal influence on the activity of some liver enzymes or binding of opioid receptors [5,6] that could modify pharmacokinetic and pharmacodynamics outcomes [7,8]. However, they are not fully translated to clinical practice [9,10] and gender issues are not deeply analysed in these observed sex-related differences [11].

While it is important to clearly distinguish between sex and gender, we also need to understand the dynamic relationship between these and other factors that influence health and well-being. Intersectional factors, such as income, social status and support, education, employment, ability, ethnicity, social and physical environments, genetics and personal health practices contribute to varied experiences and outcomes for men, women, girls, boys and gender-diverse people [12]. We need to understand the mechanisms and pathways underlying the trends we observe, and how sex and gender intersect with other factors, such as age or income to shape our overall health [13].

One of the most recent innovations in painkillers is the combination of oxycodone with naloxone (OXN), a peripherally acting μ-opioid receptor antagonist, which has helped to reduce the incidence of opioid-induced gastrointestinal adverse events (AEs) [14]. Another new opioid is tapentadol (TAP), a centrally acting μ-opioid receptor agonist and noradrenaline reuptake inhibitor with better tolerability [15] and potentially fewer pharmacological interactions [16,17]. OXN and TAP are two new-generation opioids, which apparently show a better safety profile and greater effectiveness compared to Other Prescribed Opioids (OPO) in real-world pain units (PU) [18,19]. There are some data related to the sex differences in OXN [8,20] mostly due to gastrointestinal AEs [8,20] but not as much as with TAP or comparing both opioids.

As long-term opioid treatment is challenging due to its effectiveness and safety, the fact that there is any difference between men and women should be taken into account in the pharmacotherapeutic management of chronic non-cancer pain (CNCP) patients.

In this regard, our aim was to analyse the influence of biological sex on the analgesic response in three groups of patients under OXN, TAP, or OPO, in terms of effectiveness and safety outcomes. The exploratory nature of the differences will help us to highlight sex differences for a future gender perspective analysis.

## 2. Materials and Methods

### 2.1. Study Design

A real-world cross-sectional study was conducted from April 2021 to December 2021 on CNCP outpatients treated long-term with opioids (OXN, TAP or OPO) (Targin, Mundipharma Pharmaceuticals S.L., Bahia de Pollensa Street 11, 28042 Madrid, Spain. And Palexia Grünenthal Pharma S.A., Zamenhof Street 36, 28027 Madrid, Spain). Subjects were recruited following their routine clinical visits for standard treatment at PU in the Health Department of Alicante-General Hospital, Spain. The recruitment period was from November 2014 to November 2017, since these patients were included in a previous study [21]. Upon enrolment, all patients received information on the design and purpose of the study. The Ethics Committee of the Alicante General University Hospital approved the study protocol (PI2019/108, 190715) and informed consent was obtained from all participants, allowing the use of their electronic health records (EHRs). This study is in accordance with the applicable STROBE guidelines.

### 2.2. Participants

A total of 571 CNCP patients were included under the following inclusion criteria: adult men and women (≥18 years) with a stable regimen of regular opioid prescription for more than 3 months due to CNCP, who were able to properly fill out the scales and questionnaires. There was no minimum pain score required for inclusion in the study. Opioid use was established according to medical opinion, as there is no established protocol. In addition to this, none of the patients included in this study were undergoing hormone-replacement therapy

The exclusion criteria were: the patient’s own decision to withdraw from the study, oncologic pain, cessation of allocated medication, pregnancy, due to the possible risks for both mother and baby [22], any illicit drug consumption or drug dependency, or any cognitive inability that could interfere with the proper development of the study. Furthermore, other chronic pain syndromes of unclear pathophysiology (such as fibromyalgia) or neuropathic pain syndromes (such as painful polyneuropathy, postherpetic neuralgia, trigeminal and diabetic neuralgia, peripheral nerve injury and post-stroke pain) [23] were not included in the present study. Although the study excluded these conditions associated with neuropathic pain, some of the types of pain recorded, such as low back pain itself could include a neuropathic component, having a mixed composition, and therefore, being able to associate with a neuromodulatory treatment [24,25].

### 2.3. Procedure

A consecutive sampling method was used to include ambulatory patients. Once a week researchers pre-screened candidates given early morning appointments at PU. When a patient was under TAP or OXN routine prescription and met the inclusion criteria, he/she was informed about the purpose of the study by the PU healthcare staff. The patients’ treatment was selected according to physician criteria based on the best option for the patient, due to the observational nature of the study, without any randomised procedure. The subjects concerned were attended to by the research staff and signed the informed consent paperwork always after their clinical visit, in addition to completing the questionnaires in a single visit. Subjects were then classified depending upon their standard pharmacological treatment.

The lack of randomisation led to the patients’ being either: 1/ under regular OXN or TAP prescription (at least three months before) in subjects previously naïve to opioids, 2/ switched to OXN or TAP from a different opioid (at least three months before) in patients non-naïve to opioids, or 3/ under another OPO (morphine, fentanyl, tramadol, oxycodone, buprenorphine, and hydromorphone) except OXN or TAP (at least three months before). In any case, patients were divided into each group according to the main opioid, which was the opioid with the highest Morphine Equivalent Daily Dose (MEDD). Subjects were then divided according to their sex in all three groups.

### 2.4. Data Collection

In the single study visit, data were collected using validated scales and questionnaires completed using standard clinical routines to assess pain intensity, pain relief, quality of life, and the most common AEs during pain management [26]. Patients were asked about their current pain status (at the very moment of the clinical visit), the intensity, the relief he/she feels with the opioid medication since the last visit and what was his/her current quality of life. As this is a cross-sectional study, each patient refers only to a time-point value for the scales.

Here, pain intensity, pain relief and quality of life were determined using a standardised self-reported Visual Analogue Scale (VAS). The VAS for each indicator consists of a 100 mm horizontal line ranging from 0 indicating ‘lowest’, to 100 mm indicating ‘highest’. Patients point on the line to the pain intensity, relief or quality of life they experience. Likert pain intensity and relief scales were also registered (4 = extremely intense, 3 = intense, 2 = moderate, 1 = mild, 0 = none). Greater pain relief was defined as clinically significant when there is an increase in relief or decrease in VAS pain intensity for 20 mm [27,28].

Additionally, the percentage of Emergency Department (ED) visits, hospitalisations, or drug changes due to pain or other causes since the last clinical visit was registered due to patient responses. Usually, PU visits were organised every three months. Prescription changes along the previous month included: 1) Change in any dosage, 2) product or generic brand switch, 3) stopping medication or nonadherence, and 4) starting a new medication [29]. Demographic data, pain history, drug use and medical history were completed from EHRs.

### 2.5. Drug Prescription and Adverse Events

In all the cases, MEDD was calculated based on the total sum of all opioid prescriptions during the PU visit and conversion doses due to regular international guidelines [30,31,32].

Other analgesics (NSAIDs, acetaminophen or metamizole) as well as concomitant drugs frequently used in pain management, such as anxiolytics (benzodiazepines), were also recorded from the institution’s EHRs. Furthermore, gabapentinoids (pregabalin and gabapentin) and antidepressants (amitriptyline, and duloxetine) most used as pain coadjuvants were labelled as neuromodulating agents and adjuvants in the management of patients with chronic musculoskeletal pain [33].

For the collection of patients’ reported AEs, a questionnaire with a list of the most common Adverse Drug Reactions (ADRs, selected for being “very common” or “common” on the opioids’ Summary of Product Characteristics) [34] and a blank field to add any other AEs was collected “since the last clinical visit”. These AEs consisted of somnolence, dizziness, nausea, vomiting, constipation, itching, sexual impotence, loss of libido, weight change, headache, skin redness, dry skin, dry mouth, oedema, depression, insomnia, nervousness and loss of appetite. In addition to the questionnaire, the listed ADRs were recorded from EHRs. The clinical data of the reported AE/ADR were coded according to the Medical Dictionary for Regulatory Activities (MedDRA) and the system organ class [35].

### 2.6. Statistical Analysis

Convenience sampling was considered more likely to represent the target population. This entailed selecting participants on the basis of availability until the final sample size was achieved [36]. Propensity score matching was used as a quasi-experimental method in which we construct an artificial control group by matching each treated (TAP, OXN) unit with a non-treated unit of similar characteristics (Other opioids, OPO). Data distribution was analysed with the Kolmogorov–Smirnov test using the Lilliefors correction method. Quantitative parametric data are presented as mean ± standard deviation (SD), whilst non-parametric data and discrete variables are shown using median and interquartile range (IQR). Categorical data are expressed in percentages.

We compared sociodemographic factors, medical history, clinical characteristics, and analgesics response, by sex using χ^2^ or Fisher’s exact test for categorical variables and the t-test or Mann–Whitney U test for continuous variables depending upon their distribution. When more than two groups were involved, ANOVA/Kruskal–Wallis or chi-square tests were used for continuous or categorical variables, respectively. Multiple regressions were performed to generate a predictive risk model and to analyse the influence of the following variables: pain intensity, pain relief, quality of life and MEDD: age, pain intensity, pain relief, quality of life, MEDD, number of AEs and the use of neuromodulators, antidepressants, anxiolytics, analgesics, and NSAIDs. These were carried out depending not only on sex but also on the pharmacological group. In addition to this, the effect sizes were calculated for all the comparisons. Eta-Squared (η^2^) was used for ANOVA and Kruskal–Wallis analyses (with an effect size between 0.01 and 0.04 being a small effect, 0.06 and 0.11 intermediate and 0.14 and 0.2 a large effect), whilst for the chi-square χ^2^ the effect size was determined using the Cramer’s V method (with effect size <0.2 being a small effect, 0.2 < effect size < 0.6 being intermediate, and effect size > 0.6 being a large effect) and using Odd Ratios for AEs between study groups.

*p*-value < 0.05 were considered statistically significant. Analyses were carried out using the R software package (Version 4.0.3, the GNU project, Cambridge, MA, USA) and GraphPad Prism (version 9.0., Dotmatics Boston, MA, USA).

## 3. Results

**Figure 1:**
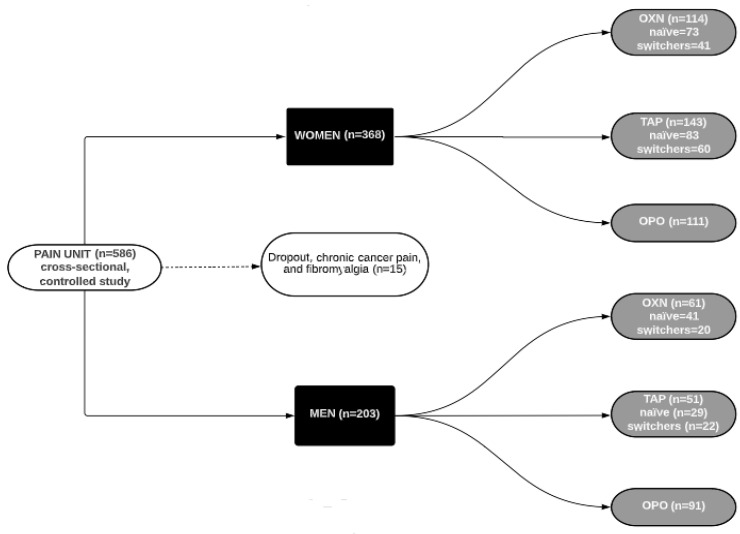
Flow chart of the study.

A total of 586 subjects were pre-screened with 7% of patients excluded (*n* = 15, mainly due to dropout, chronic cancer pain and fibromyalgia as comorbidities). Finally, 571 CNCP patients were included, of whom 64% were middle-aged women (66 (55–76) years old, all Caucasian). Here, subjects were divided according to sex: a) women (*n* = 368, 64%) divided into OXN (*n* = 114), TAP (*n* = 143) or OPO (*n* = 111); b) men (*n* = 203, 46%) divided in OXN (*n* = 61), TAP (*n* = 51) or OPO (*n* = 91) groups, as displayed in Figure 1.

Non-specific low back pain was the most common type (75%, associated with radiculopathy, spinal stenosis, or another specific spinal cause), followed by gonalgia (16%) and other musculoskeletal pain (9%, hip pain or due to other cervical joint dysfunctions). No statistically significant differences were found between opioid-naïve or switched from any other opioid regarding pain intensity, relief, and quality of life (data not shown).

### 3.1. Demographic and Clinical Outcomes

All our patients were Caucasic and residents of Spain. It is noted that five patients were foreigners, of which two were from eastern European countries, one from the Netherlands and two from South America. Globally, most of our patients suffer from moderate chronic pain (60 (35–80) mm), mild relief (30 (10–60) mm) and a moderate quality of life (50 (30–60) mm), with no differences between sexes. A summary of the characteristics of the subjects included in the study is presented in Table 1.

Results evidenced that OXN and TAP patients showed a significant three to four times lower extremely severe pain intensity and a higher mean pain relief (6–10%) than OPO (24–35%, *p* < 0.05) who referred the highest rate of ED visits due to pain. Here, OXN patients showed the highest use of hospital resources due to other causes not related to pain and prescription change due to pain, for both women and men.

#### Sex-Differences between Opioid Groups

On the whole, women were significantly three years older (66 [55–76]) than men (63 [52–73] years old, *p* < 0.05), with a difference of seven years in the OPO group (67 [52–77] vs. 60 [50–72] years old, in men).

Furthermore, a significantly greater use of hospital resources was detected in women, particularly with 8% higher ED visits and 7% prescription changes, due to other causes not related to pain (*p* < 0.05). In the opioid group, women^OXN^ visited the ED 19% more often due to pain and, together with women^TAP^ underwent 12–14% more prescription changes due to other causes. Whilst men showed a higher rate of prescription change but concretely due to pain (women^OXN^ 38% vs. 49% men^OXN^, *p* < 0.05).

### 3.2. Pharmacology Variables

A summary of pain therapy can be seen in Table 2 and Figure S1 of the Supplementary Material.

#### Sex-Differences between Opioid Groups

Higher MEDD and coadjuvant use were demonstrated for the OXN group. For the rest, data showed that women were prescribed similarly to men except for a significant 10% higher prescription rate of benzodiazepines (OR (95% CI) = 1.6 (1.1–2.3), *p* < 0.05).

Due to each opioid group, women^OXN^ produce the highest MEDD (127 mg/day, *p* < 0.05), double the prescription of benzodiazepines (46% vs. 22% men^OXN^, *p* < 0.01), and 15% less use of pregabalin (34% vs. 49% men^OXN^, *p* < 0.05). Furthermore, the OPO group showed the highest consumption of analgesics, above all 49% men^OPO^ and tramadol for 45–55% for both sexes. On the other hand, women^TAP^ required the lowest MEDD requirements (88 ± 88 mg/day) and an 11% higher use of tramadol than men^TAP^.

### 3.3. Safety Profile

Drug tolerability is shown in Table 3 and Figure S1 of the Supplementary Material. Incidence rate of five AEs/patient was shown, with the most frequents disorders being: 22% psychiatric (40% nervousness, 29% insomnia, 31% depression), 21% nervous (38% somnolence, 30% headache, 32% dizziness) and 16% gastrointestinal (62.6% constipation, 27% nausea, 10% vomiting). In total, 192 ADRs were notified (ratio of 16 AEs: 1ADR) without differences between sexes (data not shown).

#### Sex-Differences between Opioid Groups

Here, women referred to a significant higher number of AEs in all three opioid groups compared to men, specifically due to a greater frequency of dry skin, 17% (OR (95% CI = 2.22 (1.42–3.44)), weight change, 12% (40% vs. 28%, *p* < 0.001, OR (95% CI) = 2.08 (1.32–3.25)), pruritus, 9% (OR (95% CI) = 1.72 (1.11–2.65)), and headache, 8% (OR (95% CI) = 1.45 (0.99–2.13)). Whilst men developed higher frequencies of loss of libido, 12% (OR (95% CI) = 1.93 (1.22–3.04)) and 7% more sexual impotence (OR (95% CI) = 2.77 (1.53–5.01)) than women.

In relation to differences in each opioid group, OXN showed the highest AEs/patient, especially in females (OXN 6 [4–10] vs. TAP 5 [3–8] vs. OPO 5 [2–7], *p* < 0.01). Here, women^OXN^ reported the highest frequency of constipation, 71% (OXN vs, OPO, OR (95% CI) = 2.47 [1.37–4.39]; OXN vs. TAP, OR (95%CI) = 2.46 [1.45–4.15]) and 48% weight change (OXN vs. OPO, OR (95% CI) = 2.49 [1.35–4.38]; OXN vs. TAP, OR (95% CI) = 1.28 [0.78–2.10]). By contrast, men referred to more sexual AEs in all three groups than women, due to a significantly higher 9–20% sexual impotence and 10–15% loss of libido. In the same line, men^OXN^ AEs were higher than other men (TAP 4 [1–6] vs. OXN 6 [3–8] vs. OPO 4 [2–8], *p* < 0.01).

### 3.4. Multiple Linear and Logistic Regressions

Overall, pain intensity, relief, and quality of life were mutually influencing factors, as can be seen in Tables S1 and S2 of the Supplementary Material.

Here, in women, pain relief, and quality of life were negatively correlated with pain intensity, whilst the number of AEs and use of neuromodulators (pregabalin and gabapentin) was positively correlated with pain intensity, so these factors were predictive values of pain intensity in women (R^2^ = 0.38). On the other hand, in men, all these factors were also predictive values of pain intensity (R^2^ = 0.32), but the use of neuromodulators was inversely predictive since they were negatively correlated with pain intensity in men.

For instance, the use of anxiolytics was correlated with a higher pain relief in women^OXN^ (β = 0.04, *p* < 0.001, R^2^ = 0.36) and men^OXN^ (β = 2.29, *p* = 0.003, R^2^ = 0.46). In women^TAP^, pain relief (β = −0.39, *p* > 0.001), number of AEs, and quality-of-life (β = −0.332, *p* < 0.001), could predict the 46% of variance of pain intensity.

In addition to this, logistic regressions were carried out so as to analyze the influence of pregabalin prescription on sexual impotence and loss of libido incidence, since this anticonvulsant has been widely associated with sexual AEs. In this way, the dependent variables were the incidence of sexual impotence and loss of libido, while the independent variables were sex and the pregabalin’s use. Firstly, in the TAP group, neither sex nor pregabalin use showed a significant association with the frequency of sexual impotence or loss of libido. Meanwhile, in the OXN group, we found how male sex was associated with sexual impotence and loss of libido; in contrast, the use of pregabalin did not show a significant association. The regression equations were the following: Sexual impotence = −1.33 + (−2.35 × male sex (codified as 1)) *p* < 0,05; loss of libido = −0.644 + (−0.775 × male sex), *p* < 0.05. Finally, in the OPO group, loss of libido was associated with male sex but not with pregabalin prescription and showing the next equation: Loss of libido = 0.986 + (−1.02 × sex), *p* < 0.05. With these results, we could not find an association of sexual AEs with pregabalin prescription, but the male sex showed a higher frequency of sexual AEs in all three groups.

## 4. Discussion

Sex differences were found due significantly poorer tolerability and higher hospital resources in females, even worse in OXN female users who required the highest MEDD and benzodiazepine use. Other sex differences were found in previous studies [3,37] related to the older mean female age of up to seven years from males in the OPO group. These results together with other clinical outcomes (pain aetiology, psychiatric and other comorbidities, or co-medications use), and a gender perspective (socially constructed roles, behaviours, expressions and identities) could help us to understand the nature of these biological differences. Expanded development and application of methods and measures that facilitate new understandings of how sex and gender influence health, are needed [13].

According to previous studies, innovative opioids show better effectiveness than routine opioids [18,38]. In our results, there were clinically significant improvements in pain relief in the OXN and TAP groups that achieved a lower frequency of extremely severe pain compared with the OPO group. Concerning the safety profile, our data evidenced a worse tolerability pattern in the OXN group, especially in females. This could be attributed to the higher MEDD observed in this group or the higher co-prescription with benzodiazepines [39]. The latter increase, despite the lack of diagnostic data, was observed even if the frequency of nervousness and depression were reported similarly among both sexes, especially when benzodiazepines use is growing across Europe and above all in Spain [40,41]. Some studies have detected that the use of benzodiazepines grows as patients age, and especially in women [42]. Even more, the use of anxiolytics in OXN women was correlated with an increase in pain intensity. Here, a future goal will need to evaluate the potential gender differences in the use of anxiolytics, in chronic pain as has been evidenced in other illnesses [43,44]. What is more, in the European Union countries, as in many other regions of the world, national rates of illicit substance use are lower among women than men, while rates of use of licit and illicit medications, such as benzodiazepines are higher [45]. These sex differences may play an important role in pain control and should be analysed under a gender perspective research [11] even more in terms of drug safety. In addition to this, a different pattern of Tramadol use was observed between opioid groups, above all in the OPO group. Tramadol is generally used not only as a main opioid but also as a rescue medication along with stronger opioids [46]. In this way, the higher use of OPO would be caused by including patients using tramadol in opioid treatments and patients using tramadol as a rescue medication, leading to higher use in the OPO group. This difference in use could lead to future hypotheses and the study of different patterns of use depending on the main opioid and physician criteria. It should be highlighted that this study was observational and was limited to recording the pharmacological data of patients. For this reason, these differences could be caused by the different situations of patients and physician criteria, but all this should be analysed in further studies of our unit.,

On the whole, a higher number of AEs was observed for females except for the sexual area, in line with previous evidence [3,20,47,48]. In general, women were more likely to report gastrointestinal and nervous systems issues compared with men, which can lead to the higher female hospital frequentation observed in this study. There are several factors that can also influence these differences [49,50], such as the connection between hormones [51], a different pattern of co-prescribed medications, or other gender issues [52,53] that are nowadays undetected [54]. What is more, differences between males and women in sexual side-effects highlight the need for introducing this component in analgesic AE monitoring [55].

Similarly, OXN women visited the ED more frequently due to pain compared with men and were referred to a higher prescription change than in the TAP group. This could be attributed to the differences in tolerability evidenced [29,56,57] since adverse drug events are responsible for approximately 5% of unplanned hospital admissions and women are 1.5–1.7 times more likely to develop them, compared to men [58]. In fact, in a retrospective study of PU nursery teleassistance, 80% of the phone calls due to AEs were from female patients [59]. Thus, our data suggest it has not completely offset the relationship between gender and analgesic adverse outcomes [60].

It is worth noting the differences in age evidenced for women and men. The exploratory nature of the study did not allow us to collect essential information to establish whether women had received a delayed diagnosis [61,62]. We will need to consider if this age difference is due to gender stereotype threats [63,64]. However, this older age in females could affect the quality of life due to missing out on previous opportunities for pain treatment [65].

### Limitations

There are several limitations to this study that must be considered. First and foremost, the lack of randomisation is of concern and raises questions about bias. Outpatients underwent treatment prescribed by their doctor, as well as concomitant medication to treat other pathologies, for this reason, unmeasured factors may contribute to the differences observed. Along with this, patients were randomly selected as they attended their medical visit and met the inclusion criteria. Patients from the control group were also randomly recruited as long as they were not treated with either TAP or OXN; for this reason, the groups showed these distributions, where women were always the majority as in our previous studies carried out in PU [66]. It has also to be underlined that PU visits used to be organised every 3 months, although the follow-up period was not limited, which can cause changes in the patient’s health status. In addition to this, we did not have effectiveness or safety outcomes before the routine clinical visit. In this way, clinical visits were organised every 3 months in this observational study; previous data were not collected. It should be also mentioned that the list of the adverse events included in the questionnaire used in this study did not include other less common AEs caused by opioids, such as heartburn, sweating, or diarrhoea. Patients could add any other AE noticed during the study in a blank field in the questionnaire. In further studies, a greater number of AEs should be taken into account.

It should be noted also that in this study we did not include syndromes with neuropathic pain, such as trigeminal or diabetic neuralgia or post-stroke pain. This could complicate making conclusions about the effectiveness and safety of these drugs in these syndromes, which should be deeply studied in further studies in our unit. Additionally, the large amount of non-opioid centrally acting drugs taken by patients and related to other comorbidities might have independently contributed to the observed side effects. This could introduce a bias mediated by several other variables, such as socio-demographics, that could be more relevant than pain status [26].

Furthermore, important factors were not controlled during the study, such as duration of pain, type or diagnosis of pain, psychosocial factors, or variables, such as body mass index, weight or testosterone and oestrogen levels, which could interfere with the occurrence of some AEs, such as sexual impotence and loss of libido. In addition, CNCP diagnoses were made using clinical routines, but not other objective measures and approaches. This potentially clouds the understanding of what types of non-opioid analgesics, such as duloxetine or pregabalin may be appropriate for use. In addition, a convenience sample was selected based on patients attending PU. This can affect the representativeness of the population, as there were more women and it may be difficult to find significant differences in this way. On the other hand, patients in all three groups could be taking more than one opioid, and a variety of adjuvants from different opioid combinations and/or other non-analgesics could have played an important role, which was not captured in this study. One of these effects was the higher rate of constipation in the OXN group, which is against the results of some clinical assays. In this way, there are no validated tests to identify the causative agent of constipation and defecation disorders as manometric, neurophysiologic, and radiologic techniques [67]. This should be addressed in further studies from our unit.

It should also be noted that patients with psychiatric morbidity and use of illicit drugs or medical cannabinoids were excluded from participating in the study, albeit this was only controlled at the inclusion visit, which could interfere with the effectiveness and safety outcomes. In addition, MEDD was not adjusted according to the body weight of the participants; opioid dose, dose escalation and reduction were conducted according to the criteria of the physician and MEDD was calculated following regular international guidelines. In this situation, they could have independently influenced the side effects recorded and should be considered in future studies.

## 5. Conclusions

Sex differences were observed related to drug prescription, due to higher opioid, and benzodiazepine use and more prescription changes in women—especially in the OXN group (102 mg/day and 39%, respectively)—a different side-effect pattern—especially the 57% constipation in the OXN group and 16% higher male sexual AEs—higher numbers of female emergency department visits. Our understanding of sex and gender and how they intersect with other factors will continue to evolve as research advances, especially due to the impact on female pain relief and quality of life.

## Figures and Tables

**Table 1:** Demographic, clinical, and pharmacological data of women and men of the total population.

	Women (*n* = 368)	Men (*n* = 203)	OXN		TAP		OPO	
			Women (*n* = 114)	Men (*n* = 61)	Women (*n* = 143)	Men (*n* = 51)	Women (*n* = 111)	Men (*n* = 91)
**Age**	**66 [55–76] +**	63 [52–73]	67 [55–76] 65 ± 13	62 [53–72] 62 ± 14	66 [54–76] 65 ± 14	67 [54–77] 66 ± 14	67 [52–77] 64 ± 15	60 [50–72] 61 ± 14
**Pain intensity**	69 [40–80]	70 [40–80]	70 [50–80]	70 [50–80] *	60 [50–80]	67.5 [40–80] *	60 [30–80]	70 [40–90] *
**Pain relief**	38 [10–60]	30 [0–52]	**40 [10–60]** *	**50 [10–60]** *^#^	**40 [20–60]** *	30 [0–60]	27 [0–55]	20 [0–50]
**Likert pain intensity**								
None	5	6	4	2	4	11	8	7
Mild	11	7	15	9	16	15	2	4
Moderate	28	28	27	34	27	26	33	26
Severe	42	37	**47** *	**49** *	43	41 *	33	26
Extremely severe	14	22	**7** *	**6** *	**10** *	7 *	24	35
**Likert pain relief**								
None	21	24	20	24	22	27	21	19
Mild	26	31	32	33	21	35	21	20
Moderate	38	33	35	35	41	23	36	38
Severe	11	10	10	6	11	12	17	15
Extremely	4	4	3	2	4	4	5	8
**EuroQol (0–100 mm)**	50 [30–60]	50 [30–60]	50 [27.5–60]	50 [30–60]	50 [30–60]	50 [30–67.5]	50 [27–65]	50 [22–66]
**Due to pain**								
Emergency department visit	21	19	**35 +**	16	17	**13** *	29	34
Hospital admission	6	7	6	10	5	0	8	9
Prescription change	30	34	38	**49** *^#^	28	30	25	27
**Due to other causes**								
Emergency department visit	**27 +**	19	30	17	30	18	26	21
Hospital admission	14	12	**23** *^#^	20	10	8	10	10
Prescription change	**22 +**	15	30 +*	18	**22 +**	8	13	18

Note: + denotes *p* < 0.05 comparing women vs. men in total or in the same pharmacological group. The highest value is in bold font and grey. Categorical variables were analysed with chi-square test and Fisher’s exact test, continuous variables were analysed with t-test or Mann–Whitney test depending on the distribution; when 3 groups were involved, they were analysed with ANOVA or Kruskal–Wallis tests. * *p* < 0.05. comparing women or men of different groups (OPO, TAP, and OXN). # *p* < 0.05 comparing TAP and OXN.

**Table 2:** Analgesic drug prescription depending on group and sex.

Pain Medication (%)	Women (*n* = 368)	Men (*n* = 203)	OXN		TAP		OPO	
			Women (*n* = 114)	Men (*n* = 61)	Women (*n* = 143)	Men (*n* = 51)	Women (*n* = 111)	Men (*n* = 91)
Analgesic	34	38	32	27	38	30	31	49 *++
Tramadol	12	13	8	6	**20 +^#^**	9	45 **	55 **
NSAIDs	24	28	13	11	12	13	11	13
**Opioids** (%)								
MEDD (mg/day)	102 ± 96	96 ± 100	127 ± 103 +**	117 ± 122 *	88 ± 88	90 ± 88	92 ± 92	85 ± 86
Fentanyl transdermal	19	22	16	16	8	6	37 **	36 **
Oxycodone	3	5	2	2	2	4	11 *	8
Morphine	6	9	2	3	4	11	7 **	11
Buprenorphine	5	2	3	2	1	0	14 +**	4
Hydromorphone	3	1	2	0	1	0	5 *	2
**Neuromodulators** (%)								
Pregabalin	29	33	34	49 +** ^#^	23	28	32	25
Gabapentin	13	12	13	13	10	11	16	11
Duloxetine	18	20	25 ^#^	**24** *	14	20	15	18
Benzodiazepines	**39 +**	29	46 ++* ^#^	22	32 ^#^	30	40	42 *

Note: MEDD: Morphine Equivalent Daily Dose. + denotes *p* < 0.05 comparing women vs. men in total or in the same pharmacological group ++ denotes *p* < 0.01 comparing women vs. men. The highest value is in bold font and grey. * *p* < 0.05 ** *p* < 0.01 comparing women or men of different groups (OPO, TAP, and OXN). # *p* < 0.05 comparing TAP and OXN.

**Table 3:** Percentage of patients with adverse events of patients (AEs) self-reported in in women and men of other prescribed opioids (OPO), tapentadol (TAP) and oxycodone/naloxone (OXN) cases groups.

	Women (*n* = 368)	Men (*n* = 203)	OXN		TAP		OPO	
			Women (*n* = 114)	Men (*n* = 61)	Women (*n* = 143)	Men (*n* = 51)	Women (*n* = 111)	Men (*n* = 91)
**Total (%)**	**6 [3–8] +**	5 [2–8]	6 [4–10] **^##^	6 [3–8] *^#^	5 [3–8] +**	4 [1–6]	5 [2–7]	4 [2–8]
**Somnolence**	43	41	48	**48** *	39	45	43	33
**Dizziness**	37	30	40	31	37	26	34	32
**Nausea**	24	22	31	25 ^#^	22	13	20	26 *
**Vomits**	10	7	13	7	8	4	9	9
**Constipation**	**57 +**	49	**71** **^##^	**63** *^#^	50	43	49	43
**Redness skin**	17	13	**26** *^#^	**21** *^#^	12	9	**14 +**	7
**Sexual impotence**	9	**16 +**	3	23 +*	10	19 *	13 *	10
**Loss of libido**	18	**31 ++**	19	**34 +**	22	32	12	**27 +**
**Weight change**	**40 ++**	28	48 +*	23	42	30^#^	33	23
**Headache**	**36 +**	28	38	**39** *	**36 +**	21^#^	34	21
**Itching**	18	12	30	18	28	15	24	20
**Dry skin**	**43 ++**	26	51 +*	31	**43 +**	23	34	31
**Xerostomia (dry mouth)**	65	61	72	62	64	62	59	60
**Edema**	17	13	**23** *	18	**19** *	13	9	9
**Depression**	36	34	38	38	37	23	33	37
**Insomnia**	33	32	23	**18** *	**35** *^#^	**38** *^#^	23	31
**Nervousness**	45	44	**54** *	52	44	34^#^	37	43
**Lack of appetite**	31	25	33	25	33	21	25	28

Note: + denotes *p* < 0.05 comparing women vs. men in total or in the same pharmacological group ++ denotes *p* < 0.01 comparing women vs. men. The highest value is in bold font and grey. * *p* < 0.05 ** *p* < 0.01 comparing women or men of different groups (OPO, TAP, and OXN). # *p* < 0.05 comparing TAP and OXN, ## *p* < 0.01 comparing TAP vs. OXN.

## Data Availability

The data presented in this study are available on request from the corresponding author. The data are not publicly available because they contain clinical data of patients.

## Supplementary Materials

The following supporting information can be downloaded at: https://www.mdpi.com/article/10.3390/biomedicines10102468/s1. Figure S1: Morphine Equivalent Daily Dose (MEDD) and Number of Adverse Events (AEs) according to the group and depending on sex. Table S1: Multiple Linear regression of descriptive, clinical, and pharmacological parameters with VAS pain, VAS relief, quality of life, and Morphine Equivalent Daily Dose as dependent variables in women and men groups. Table S2: Multiple Linear regression of descriptive, clinical, and pharmacological parameters with VAS pain, VAS relief, and quality of life as dependent variables in women and men depending upon the pharmacological treatment.

## References

[1] Pisanu C., Franconi F., Gessa G.L., Mameli S., Pinasu G.M., Campesi I., Leggio L., Agabio R. (2019). Sex Differences in the Response to Opioids for Pain Relief: A Systematic Review and Meta-Analysis. Pharmacol. Res..

[2] Kiely K.M., Brady B., Byles J. (2019). Gender, Mental Health and Ageing. Maturitas.

[3] Margarit C., Roca R., Inda M.D.M., Muriel J., Ballester P., Flor A., Morales D., Peiró A.M. (2020). Gender Bias and Genotype Influence on Opioid Safety Profile in Chronic Low Back Pain. Clin. J. Pain.

[4] Beery T.A. (1995). Gender Bias in the Diagnosis and Treatment of Coronary Artery Disease. Heart Lung.

[5] Lopes G.S., Bielinski S.J., Moyer A.M., Black Lii J.L., Jacobson D.J., Jiang R., Larson N.B., St Sauver J.L. (2020). Sex Differences in Associations Between CYP2D6 Phenotypes and Response to Opioid Analgesics. Pharmgenomics Pers. Med..

[6] Samulowitz A., Gremyr I., Eriksson E., Hensing G. (2018). “Brave Men” and “Emotional Women”: A Theory-Guided Literature Review on Gender Bias in Health Care and Gendered Norms towards Patients with Chronic Pain. Pain Res. Manag..

[7] Linares O.A., Daly D., Linares A.D., Stefanovski D., Boston R.C. (2014). Personalized Oxycodone Dosing: Using Pharmacogenetic Testing and Clinical Pharmacokinetics to Reduce Toxicity Risk and Increase Effectiveness. Pain Med..

[8] Graziani M., Nisticò R. (2016). Gender Difference in Prescription Opioid Abuse: A Focus on Oxycodone and Hydrocodone. Pharmacol. Res..

[9] Mogil J.S., Bailey A.L. (2010). Sex and Gender Differences in Pain and Analgesia. Prog. Brain Res..

[10] Wiesenfeld-Hallin Z. (2005). Sex Differences in Pain Perception. Gend. Med..

[11] Pieretti S., Di Giannuario A., Di Giovannandrea R., Marzoli F., Piccaro G., Minosi P., Aloisi A.M. (2016). Gender Differences in Pain and Its Relief. Ann. Ist. Super. Sanita.

[12] Dao T.T.T., LeResche L. (2000). Gender Differences in Pain. J. Orofac. Pain.

[13] Canadian Institutes of Health Research (2018). Science Is Better with Sex and Gender. Strategic Plan 2018–2023.

[14] Smith K., Hopp M., Mundin G., Bond S., Bailey P., Woodward J., Palaniappan K., Church A., Limb M., Connor A. (2011). Naloxone as Part of a Prolonged Release Oxycodone/Naloxone Combination Reduces Oxycodone-Induced Slowing of Gastrointestinal Transit in Healthy Volunteers. Expert Opin. Investig. Drugs.

[15] Langford R.M., Knaggs R., Farquhar-Smith P., Dickenson A.H. (2016). Is Tapentadol Different from Classical Opioids? A Review of the Evidence. Br. J. Pain.

[16] Abeyaratne C., Lalic S., Bell J.S., Ilomäki J. (2018). Spontaneously Reported Adverse Drug Events Related to Tapentadol and Oxycodone/Naloxone in Australia. Ther. Adv. Drug Saf..

[17] Amato D., Kruyer A., Samaha A.-N., Heinz A. (2019). Hypofunctional Dopamine Uptake and Antipsychotic Treatment-Resistant Schizophrenia. Front. Psychiatry.

[18] Polati E., Canonico P.L., Schweiger V., Collino M. (2019). Tapentadol: An Overview of the Safety Profile. J. Pain Res..

[19] Vondrackova D., Leyendecker P., Meissner W., Hopp M., Szombati I., Hermanns K., Ruckes C., Weber S., Grothe B., Fleischer W. (2008). Analgesic Efficacy and Safety of Oxycodone in Combination with Naloxone as Prolonged Release Tablets in Patients with Moderate to Severe Chronic Pain. J. Pain.

[20] Lopes G.S., Bielinski S., Moyer A.M., Jacobson D.J., Wang L., Jiang R., Larson N.B., Miller V.M., Zhu Y., Cavanaugh D.C. (2021). Sex Differences in Type and Occurrence of Adverse Reactions to Opioid Analgesics: A Retrospective Cohort Study. BMJ Open.

[21] Barrachina J., Margarit C., Muriel J., López-Gil S., López-Gil V., Vara-González A., Planelles B., Inda M.-M., Morales D., Peiró A.M. (2022). Oxycodone/Naloxone vs. Tapentadol in Real-World Chronic Non-Cancer Pain Management: An Observational and Pharmacogenetic Study. Sci. Rep..

[22] European Monitoring Centre for Drugs and Drug Addiction Pregnancy and Opioid Use: Strategies for Treatment. https://www.emcdda.europa.eu/publications/emcdda-papers/pregnacy-opioid-use_en.

[23] Baron R., Binder A., Wasner G. (2010). Neuropathic Pain: Diagnosis, Pathophysiological Mechanisms, and Treatment. Lancet. Neurol..

[24] Costigan M., Scholz J., Woolf C.J. (2009). Neuropathic Pain: A Maladaptive Response of the Nervous System to Damage. Annu. Rev. Neurosci..

[25] Colloca L., Ludman T., Bouhassira D., Baron R., Dickenson A.H., Yarnitsky D., Freeman R., Truini A., Attal N., Finnerup N.B. (2017). Neuropathic Pain. Nat. Rev. Dis. Prim..

[26] Barrachina J., Muriel J., Margarit C., Planelles B., Ballester P., Richart-Martínez M., Cutillas E., Zandonai T., Morales D., Peiró A.M. (2021). Global Pain State Questionnaire: Reliability, Validity, and Gender Gap. Arch. Intern. Med. Res..

[27] Bird S.B., Dickson E.W. (2001). Clinically Significant Changes in Pain along the Visual Analog Scale. Ann. Emerg. Med..

[28] Kelly A.M. (2001). The Minimum Clinically Significant Difference in Visual Analogue Scale Pain Score Does Not Differ with Severity of Pain. Emerg. Med. J..

[29] Sino C.G., Stuffken R., Heerdink E.R., Schuurmans M.J., Souverein P.C., Egberts T.C. (2013). The Association between Prescription Change Frequency, Chronic Disease Score and Hospital Admissions: A Case Control Study. BMC Pharmacol. Toxicol..

[30] Mercadante S., Caraceni A. (2011). Conversion Ratios for Opioid Switching in the Treatment of Cancer Pain: A Systematic Review. Palliat. Med..

[31] Mercadante S., Porzio G., Aielli F., Adile C., Verna L., Ficorella C., Giarratano A., Casuccio A. (2013). Opioid Switching from and to Tapentadol Extended Release in Cancer Patients: Conversion Ratio with Other Opioids. Curr. Med. Res. Opin..

[32] Faculty of Pain Medicine of the Royal College of Anaesthetists Dose Equivalents and Changing Opioids. https://fpm.ac.uk/opioids-aware-structured-approach-opioid-prescribing/dose-equivalents-and-changing-opioids.

[33] Richards B.L., Whittle S.L., Buchbinder R. (2012). Neuromodulators for Pain Management in Rheumatoid Arthritis. Cochrane Database Syst. Rev..

[34] Boiarkina A., Potapov A. (2014). Impact of Genetic Factors on Severity of Side Effects of Opioids in Patients after Major Surgical Interventions. Klin. Khirurhiia.

[35] Spanish Agency of Medicines and Health Products (AEMPS-CIMA) Online Information Center of Medicines of Spanish Agency of Medicines and Health Products (AEMPS-CIMA). https://cima.aemps.es/cima/publico/home.html.

[36] Mathieson K. (2014). Making Sense of Biostatistics: Types of Nonprobability Sampling. J. Clin. Res. Best Pract..

[37] Planelles B., Margarit C., Inda M.-D.-M., Ballester P., Muriel J., Barrachina J., Ajo R., Esteban M.D., Peiró A.M. (2020). Gender Based Differences, Pharmacogenetics and Adverse Events in Chronic Pain Management. Pharmacogenomics J..

[38] Jovey R.D., Ennis J., Gardner-Nix J., Goldman B., Hays H., Lynch M., Moulin D., Canadian Pain Society (2019). Use of Opioid Analgesics for the Treatment of Chronic Noncancer Pain—A Consensus Statement and Guidelines from the Canadian Pain Society, 2002. Pain Pract..

[39] Hawkins E.J., Malte C.A., Grossbard J.R., Saxon A.J. (2015). Prevalence and Trends of Concurrent Opioid Analgesic and Benzodiazepine Use Among Veterans Affairs Patients with Post-Traumatic Stress Disorder, 2003-2011. Pain Med..

[40] United Nations (2020). Informe de La Junta Internacional de Fiscalización de Estupefacientes Correspondientes a 2019.

[41] Sistema Nacional de Salud, Ministerio de Sanidad (2018). Prestación Farmacéutica En El Sistema Nacional de Salud. Informe Anual Del Sistema Nacional de Salud 2018.

[42] Huerta C., Abbing-Karahagopian V., Requena G., Oliva B., Alvarez Y., Gardarsdottir H., Miret M., Schneider C., Gil M., Souverein P.C. (2016). Exposure to Benzodiazepines (Anxiolytics, Hypnotics and Related Drugs) in Seven European Electronic Healthcare Databases: A Cross-National Descriptive Study from the PROTECT-EU Project. Pharmacoepidemiol. Drug Saf..

[43] Karanti A., Bobeck C., Osterman M., Kardell M., Tidemalm D., Runeson B., Lichtenstein P., Landén M. (2015). Gender Differences in the Treatment of Patients with Bipolar Disorder: A Study of 7354 Patients. J. Affect. Disord..

[44] Boyd A., Van de Velde S., Pivette M., ten Have M., Florescu S., O’Neill S., Caldas-de-Almeida J.M., Vilagut G., Haro J.M., Alonso J. (2015). Gender Differences in Psychotropic Use across Europe: Results from a Large Cross-Sectional, Population-Based Study. Eur. Psychiatry.

[45] United Nations—Office on Drugs and Crime (2004). Substance Abuse Treatment and Care for Women: Case Studies and Lessons Learned.

[46] Chung J.W.Y., Zeng Y., Wong T.K.S. (2013). Drug Therapy for the Treatment of Chronic Nonspecific Low Back Pain: Systematic Review and Meta-Analysis. Pain Physician.

[47] Craft R.M. (2003). Sex Differences in Opioid Analgesia: “From Mouse to Man”. Clin. J. Pain.

[48] Muriel J., Margarit C., Barrachina J., Ballester P., Flor A., Morales D., Horga J.F., Fernández E., Peiró A.M. (2019). Pharmacogenetics and Prediction of Adverse Events in Prescription Opioid Use Disorder Patients. Basic Clin. Pharmacol. Toxicol..

[49] Centers for Disease Control and Prevention Understanding the Epidemic|CDC’s Response to the Opioid Overdose Epidemic|CDC. https://www.cdc.gov/opioids/basics/epidemic.html.

[50] U.S. Department of Health and Human Services (2017). Final Report: Opioid Use, Misuse, and Overdose in Women.

[51] Nikolov V., Petkova M. (2010). Pain Sensitivity among Women with Low Estrogen Levels. Procedia Soc. Behav. Sci..

[52] Claréus B., Renström E.A. (2019). Physicians’ Gender Bias in the Diagnostic Assessment of Medically Unexplained Symptoms and Its Effect on Patient-Physician Relations. Scand. J. Psychol..

[53] Campesi I., Montella A., Seghieri G., Franconi F. (2021). The Person’s Care Requires a Sex and Gender Approach. J. Clin. Med..

[54] Ruiz-Cantero M.-T., Blasco-Blasco M., Chilet-Rosell E., Peiró A.M., Ruiz-Cantero M.-T., Blasco-Blasco M., Chilet-Rosell E., Peiró A.M. (2020). Sesgos de Género En El Esfuerzo Terapéutico: De La Investigación a La Atención Sanitaria. Farm. Hosp..

[55] Gombert M., Ballester P., Segura A., Peiró A.M. (2021). Introducing Sexual Dysfunction in Mental Care. Expert Opin. Drug Saf..

[56] Loikas D., Wettermark B., Von Euler M., Bergman U., Schenck-Gustafsson K. (2013). Differences in Drug Utilisation between Men and Women: A Cross-Sectional Analysis of All Dispensed Drugs in Sweden. BMJ Open.

[57] Orlando V., Mucherino S., Guarino I., Guerriero F., Trama U., Menditto E. (2020). Gender Differences in Medication Use: A Drug Utilization Study Based on Real World Data. Int. J. Environ. Res. Public Health.

[58] Hendriksen L.C., van der Linden P.D., Lagro-Janssen A.L.M., van den Bemt P.M.L.A., Siiskonen S.J., Teichert M., Kuiper J.G., Herings R.M.C., Stricker B.H., Visser L.E. (2021). Sex Differences Associated with Adverse Drug Reactions Resulting in Hospital Admissions. Biol. Sex Differ..

[59] López-Ramal A. (2022). Comunicación Telefónica de Eventos Adversos En La Terapéutica Del Dolor: Diferencias Por Sexo y Genética.

[60] Duvernoy C.S., Smith D.E., Manohar P., Schaefer A., Kline-Rogers E., Share D., McNamara R., Gurm H.S., Moscucci M. (2010). Gender Differences in Adverse Outcomes after Contemporary Percutaneous Coronary Intervention: An Analysis from the Blue Cross Blue Shield of Michigan Cardiovascular Consortium (BMC2) Percutaneous Coronary Intervention Registry. Am. Heart J..

[61] Ruiz-Cantero M.T., Verdú-Delgado M. (2004). Sesgo de Género En El Esfuerzo Terapéutico. Gac. Sanit..

[62] Husby G.K., Haugen R.S., Moen M.H. (2003). Diagnostic Delay in Women with Pain and Endometriosis. Acta Obstet. Gynecol. Scand..

[63] Schäfer G., Prkachin K.M., Kaseweter K.A., de Williams A.C. (2016). Health Care Providers’ Judgments in Chronic Pain: The Influence of Gender and Trustworthiness. Pain.

[64] Nguyen R.H.N., Turner R.M., Rydell S.A., Maclehose R.F., Harlow B.L. (2013). Perceived Stereotyping and Seeking Care for Chronic Vulvar Pain. Pain Med..

[65] Tabach Apraiz A., Lorena Oyanadel M., Gutiérrez Espinoza H., Bueno Buker D., Tabach Apraiz A., Lorena Oyanadel M., Gutiérrez Espinoza H., Bueno Buker D. (2019). Correlation study between diagnostic opportunity and pain severity in patients with fibromyalgia who enter the Nononcological Chronic Pain Unit at the San Borja Arriarán Clinical Hospital. Rev. La Soc. Española Del Dolor.

[66] Planelles B., Margarit C., Ajo R., Sastre Y., Muriel J., Inda M.-D.-M., Esteban M.D., Peiró A.M. (2019). Health Benefits of an Adverse Events Reporting System for Chronic Pain Patients Using Long-Term Opioids. Acta Anaesthesiol. Scand..

[67] Rao S.S.C., Meduri K. (2011). What Is Necessary to Diagnose Constipation?. Best Pract. Res. Clin. Gastroenterol..

