# Supplementary material for: Sex Differences in Oxycodone/Naloxone vs. Tapentadol in Chronic Non-Cancer Pain: An Observational Real-World Study
Source: Biomedicines. 2022 Oct 2;10(10):2468. doi: 10.3390/biomedicines10102468 (PMC9598624; doi:10.3390/biomedicines10102468)
Supplement: Supplementary file 1 [file biomedicines-10-02468-s001.zip › biomedicines-1863642-supplementary.pdf]

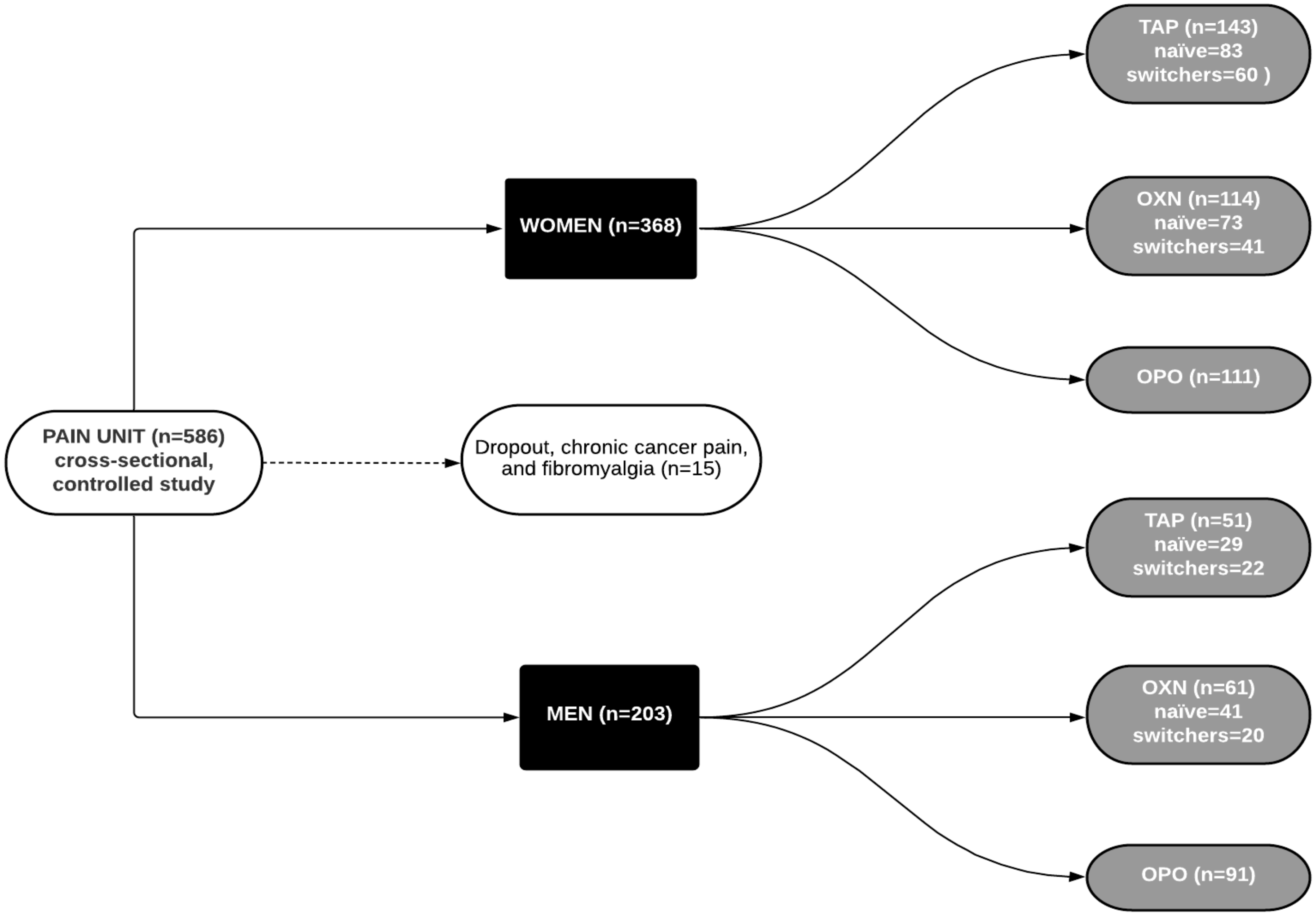
PAIN UNIT (n=586)
cross-sectional,
controlled study
WOMEN (n=368)
Dropout, chronic cancer pain,
and fibromyalgia (n=15)
MEN (n=203)
TAP (n=143)
naïve=83
switchers=60)
OXN (n=114)
naïve=73
switchers=41
OPO (n=111)
TAP (n=51)
naïve=29
switchers=22
OXN (n=61)
naïve=41
switchers=20
OPO (n=91)

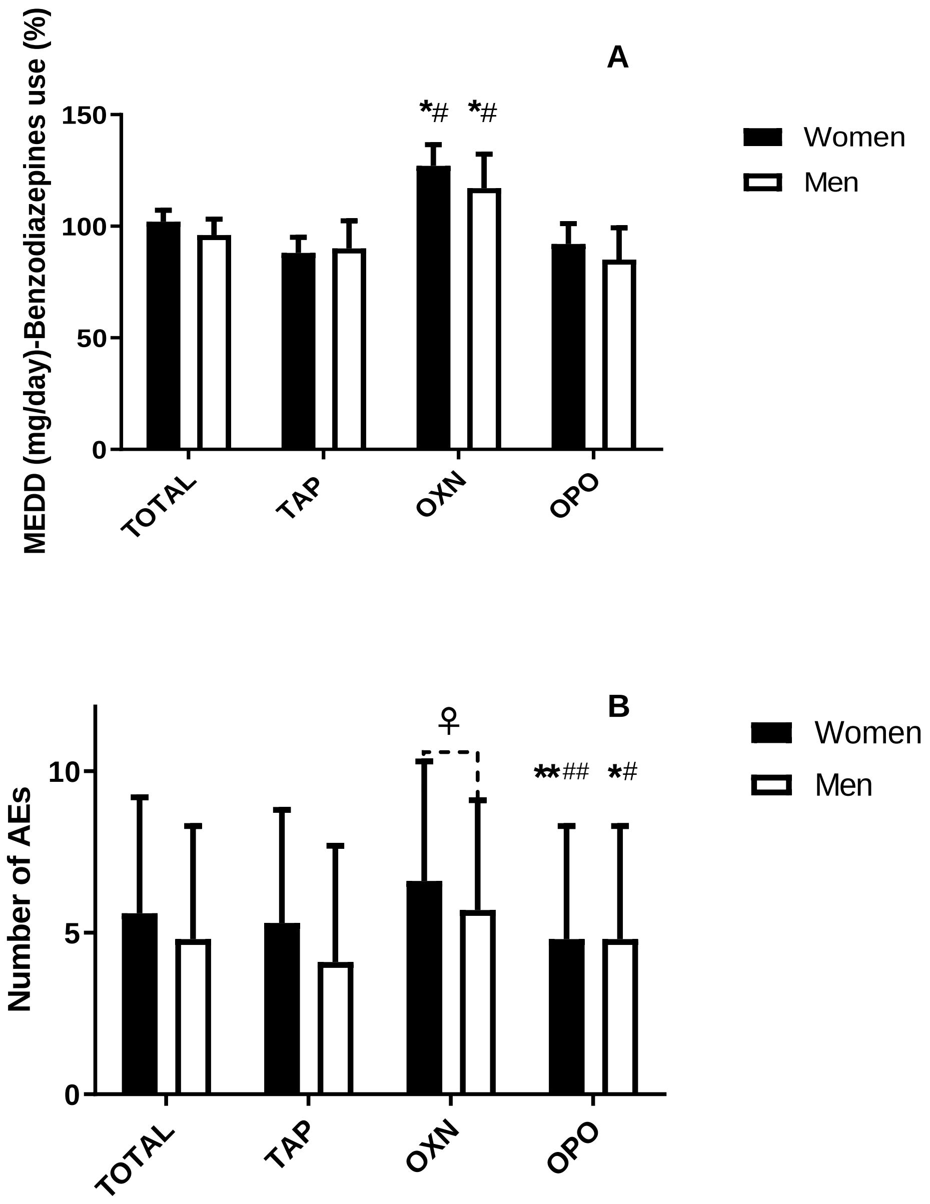

Figure S1: Morphine Equivalent Daily Dose (MEDD) and Number of Adverse Events (AEs) according to the group and depending on sex. Note: * denotes $p < 0.05$ comparing men between groups, ** denotes $p < 0.01$ comparing women between groups. denotes $p < 0.05$ comparing women with men of the same group. # Denotes $p < 0.05$ comparing TAP vs. OXN, ## denotes $p < 0.01$ comparing TAP vs. OXN.

Table S1.- Multiple Linear regression of descriptive, clinical and pharmacological parameters with VAS pain, VAS relief, quality of life, and Morphine Equivalent Daily Dose as dependent variable in women and men groups.

**Pain intensity**	**Model p-value** **R^2**	**Predictive variables**	**Beta coefficient (CI 95%)**	**p-value**
Women	<0.001 0.377	Pain relief	-0.381 (-0.47-(-0.29))	<0.001
		Quality of life	-0.021 (-0.03-0.009)	<0.001
		Number of AE	0.147 (0.07-0.22)	<0.001
		Use of Neuromodulators	0.656 (0.17-1.14)	0.008
Men	<0.001 0.326	Pain Relief	-0.373 (-0.50-(-0.24))	<0.010
		Quality of life	-0.02 (-0.04-(-0.003))	0.023
		Number of AE	0.122 (0.01- 0.23)	0.032
		Use of Neuromodulators	-0.819 (-1.6-(-0.03)	0.040
**MEDD**				
Women	0.033 0.016	Pain relief	3.215 (-0.48-6.91)	0.045
		Number of AE	3.490 (0.431-6.548)	0.025
**Pain relief**				
Men	3.069×10^{-11} 0.2441	Quality of life	0.029 (0.01-0.04)	0.001
		Pain intensity	-0.400 (-0.54-(-0.25))	1.81×10^{-7}
Women	2.2×10^{-16} 0.241	Quality of life	0.031 (0.02-04)	2.51×10^{-6}
		Pain intensity	-0.364 (-0.47(-0.26))	6.91×10^{-11}
**Quality of life**				
Women	5.5×10^{-16} 0.205	Pain relief	2.07 (1.15-2.87)	5.87×10^{-6}
		Pain intensity	-1.51 (--2.45(-0.57))	0.001
		AE	-1.13 (-1.80-(--0.45))	0.001

Men	$1.47x10^{-7}$<br>0.198	Pain relief	2.049 (0.85-3.24)	0.0008
		Pain intensity	-1.306 (-2.58(-0.02))	0.045
		Number of AE	-1 (-1.92(-0.07)	0.034
		Antidepressant	-7.14 (-13.77(-0.52)	0.034

Table S2- Multiple Linear regression of descriptive, clinical, and pharmacological parameters with VAS pain, VAS relief and quality of life as dependent variable in women and men depending upon pharmacological treatment.

Regression Equation	β Coefficient	SE	T	Sig. T	95% CI for Exp (B)		R^2	F test	p
					LL	UL			
**OPO Women Pain intensity**									
**Constant**	5.345	0.856	6.240	<0.001	3.641	7.049	0.174	9.952	<0.001
**Adverse Events**	0.309	0.090	3.416	<0.001	0.129	0.489			
**Quality of life**	-0.029	0.012	-2.328	0.022	-0.054	-0.004			
**OPO Women Pain relief**									
**Constant**	1.776	0.698	2.544	0.012	0.386	3.165	0.058	6.067	0.016
**Quality of life**	0.032	0.013	2.463	0.016	0.006	0.058			
**OPO Women Quality of life**									
**-**	-	-	-	-	-		-	-	-
**OPO Men Pain Intensity**									
**Constant**	8.198	0.672	12.187	<0.001	6.851	9.544	0.230	8.685	<0.001
**Pain relief**	-0.423	0.122	-3.471	<0.001	-0.668	-0.179			
**Analgesics**	-1.653	0.722	-2.289	0.025	-3.098	-0.207			
**OPO Men Pain relief**									
**Constant**	3.284	0.964	3.405	<0.001	1.359	5.210	0.294	14.22	<0.001
**Pain intensity**	-0.342	0.097	-3.495	<0.001	-0.537	-0.146			
**Quality of life**	0.036	0.011	3.039	0.003	0.012	0.060			
**OPO Men Quality of life**									
**Constant**	50.318	5.375	8.773	<0.001	38.85	61.78	0.316	9.552	<0.001
**Pain relief**	3.787	0.924	4.099	<0.001	1.940	5.634			
**Adverse events**	-1.687	0.760	-2.219	0.030	-3.207	-0.167			
**Neuromodulators**	-13.529	5.541	-2.242	0.017	-24.605	-2.452			
**TAP Women pain intensity**									
**Constant**	8.080	0.574	14.068	<0.001	6.940	9.216	0.465	37.41	<0.001
**Pain Relief**	-0.396	0.068	-5.814	<0.001	-0.531	-0.261			
**Quality of life**	-0.032	0.008	-3.689	<0.001	-0.050	-0.015			

**Adverse events**	0.177	0.052	3.361	<0.001	0.072	0.281			
				**TAP Women pain relief**					
**Constant**	5.877	0.882	6.662	<0.001	4.131	7.622			
**Pain intensity**	-0.522	0.085	-6.142	<0.001	-0.690	-0.354	0.362	36.93	<0.001
**Quality of life**	0.022	0.010	2.138	0.034	0.001	0.043			
				**TAP Women quality of life**					
**Constant**	58.155	6.647	8.753	<0.001	45.033	71.336			
**Pain intensity**	-3.155	0.743	-4.190	<0.001	-4.586	-1.644	0.275	24.67	<0.001
**Pain relief**	1.511	0.707	2.138	0.034	0.113	2.910			
				**TAP Men pain intensity**					
**Constant**	8.237	0.758	10.860	<0.001	6.710	9.764	0.202	11.66	0.001
**Quality of life**	-0.049	0.014	-3.415	0.001	-0.079	-0.020			
				**TAP Men pain relief**					
**Constant**	1.315	0.862	1.526	0.133	-0.419	3.051	0.137	7.302	0.009
**Quality of life**	0.049	0.016	2.702	0.009	0.011	0.078			
				**TAP Men quality of life**					
**Constant**	58.659	9.933	5.905	<0.001	38.652	78.665			
**Pain intensity**	-3.283	1.240	-2.648	0.011	-5.779	-0.786	0.253	7.635	0.001
**Pain relief**	1.992	1.134	1.756	0.061	-0.292	4.276			
				**OXN Women pain intensity**					
**Constant**	10.322	1.086	9.503	<0.001	8.170	12.475			
**Pain relief**	-0.44	0.071	-5.779	<0.001	-0.556	-0.272	0.293	15.38	<0.001
**Anxiolytics**	1.139	0.453	2.514	0.013	0.241	2.038			
**Age**	-0.043	0.016	-2.632	0.009	-0.076	-0.010			
				**OXN Women pain relief**					
**Constant**	4.684	0.851	5.499	<0.001	2.995	6.372			
**Pain intensity**	-0.481	0.089	-5.396	<0.001	-0.658	-0.304	0.361	20.55	<0.001
**Quality of life**	1.285	0.484	2.653	0.009	0.325	2.245			
**Anxiolytics**	0.040	0.010	4.017	<0.001	0.020	0.060			
				**OXN Women quality of life**					
**Constant**	46.513	5.357	8.681	<0.001	35.893	57.133	0.294	15.01	<0.001
**Pain relief**	2.620	0.650	4.025	<0.001	1.329	3.910			

**Analgesics**	9.144	4.175	2.190	0.030	0.867	17.412			
**Adverse events**	-2.910	0.542	-4.039	<0.001	-3.265	-1.115			
**OXN Men pain intensity**									
**Constant**	9.520	0.600	15.863	<0.001	8.318	10.722	0.413	13.38	<0.001
**Pain relief**	-0.535	0.087	-6.140	<0.001	-0.709	-0.360			
**Neuromodulators**	-1.564	0.569	-2.747	0.008	-2.705	-0.423			
**Anxiolytics**	1.273	0.669	1.902	0.062	-0.067	2.613			
**OXN Men pain relief**									
**Constant**	9.766	1.018	9.585	<0.001	7.726	11.807	0.458	16.12	<0.001
**Pain intensity**	-0.743	0.121	-6.140	<0.001	-0.986	-0.501			
**Neuromodulators**	-1.953	0.666	-2.932	0.004	-3.287	-0.619			
**Anxiolytics**	2.297	0.755	3.043	0.003	0.785	3.809			
**OXN Men quality of life**									
**-**	-	-	-	-	-	-	-	-	-
